# Experimental Study on the Mechanical Properties of Porcine Cartilage with Microdefect under Rolling Load

**DOI:** 10.1155/2017/2306160

**Published:** 2017-06-12

**Authors:** Yu-tao Men, Xiao-ming Li, Ling Chen, Hu Fu

**Affiliations:** ^1^Tianjin Key Laboratory of the Design and Intelligent Control of the Advanced Mechatronical System, Tianjin, China; ^2^School of Mechanical Engineering, Tianjin University of Technology, Tianjin 300384, China

## Abstract

**Objectives:**

To investigate the mechanical responses of microdefect articular cartilage under rolling load and find out the failure rule.

**Methods:**

Rolling load was applied to the porcine articular cartilage samples with rectangular notches of different depths. The displacement and strain near the notches were obtained by the noncontact digital image correlation technique.

**Results:**

The strain value and peak frequency around the notch increased; the maximum equivalent strain value could be observed at both bottom corners of the notch; the equivalent strain value first increased and then decreased at the points in the superficial and middle layers with the increase of rolling velocity; the points in the deep layer were less affected by rolling velocity; the equivalent strain value of the points in the superficial layer declined after rising with the increase of defect depth, while a decreased trend could be found for the points in the middle and deep layers.

**Conclusions:**

The shear strain, which rose with the increase in defect depth, was the main factor in cartilage destruction. The cartilage tended to be destructed firstly at the bottom corner of the defect. Rolling velocity showed significant effects on superficial and middle layers. Cartilage had the ability to resist destruction.

## 1. Introduction

The articular cartilage is an important part of the human bone joints, which plays critical roles in reducing vibration and bone protection in daily activities of the human body. Studies suggested that the articular cartilage has a quite complex composition and specific material properties like nonlinearity and viscoelasty [1]. The articular cartilage may be damaged to varying degrees due to excessive exercise or other sudden causes [2–4]. However, lots of patients present no symptom in the early stage of cartilage damage, and if let to accumulate, it could evolve into osteoarthritis which will then affect daily life [5, 6].

The articular cartilage can rarely be repaired if damaged; due to the absence of blood vessel, this usually results in osteoarthritis. Therefore, the study on mechanical properties of cartilage with the defect has started drawing attention in the scientific literature. Repeated load deformation can cause fatigue wear, which alters the mechanical properties of the cartilage. Generally, the greater the surface roughness, the faster the wear is. Delamination damage is the major form of damage for a cartilage under a friction load [7]. Damage will change the mechanical properties of the cartilage [8], causing reduced rigidity [9, 10] or increased permeability [11] for instances. Cyclic loading experiment of cartilage showed that cyclic loading at 7–17 MPa could lead to cartilage cell death but not to damage on the surface structure [12]. By using *α*-chymotrypsin to label collagenous fibers, the cartilage was found to be deeply stained and marked fibrosis could be observe under acyclic load of 5 MPa for 20 minutes [13]. Gratz et al. [14] have explored changes in a notch angle in full-thickness-defected cartilage under a compressive load. It was found that the closed injury was more likely to slip than the open one. The experimental results of Stok and Oloyede [15, 16] showed that the crack propagation mechanism of the cartilage crack was quite different from the open mode of traditional fracture mechanics. Dabiri and Li [17] analyzed data from a knee joint model and found a gradually decreased osmotic pressure but an increased shear strain with the increase in cartilage degradation. Based on a 3-D ankle model, Hua et al. [18] observed that the peak stress values increased significantly as the articular cartilage defect area increased; joint functions will be remarkably affected when defect diameter exceeded 11 mm in the distal tibial articular cartilage. Hosseini et al. [19] showed the interactions between softening of bone matrix and damage of fibers. Numerical model also proved that the damage grew preferentially along the tangent direction of the fibers. Fibers played an important role in cartilage construct [20].

The distribution mode of collagen fibers in the cartilage plays a key role in the mechanical properties of cartilage. Based on the distribution and arrangement mode of the collagen fibers, the articular cartilage can be roughly divided into three layers [21]: superficial (tangential layer), middle (transitional layer), and deep layers (radiation). It was reported that changes in the displacement derived from different compressive levels; the loading velocity and the loading times gradually decreased from the surface to the deep layer [22]; and the superficial layer has a vital role in maintaining the biomechanical properties of the cartilage.

Previous studies have focused on the analysis of the mechanical properties of intact cartilage; however, various degrees of articular cartilage damages can be observed even in the early stage of osteoarthritis [23]. It often takes more than ten years or even decades from the initial damage to the loss of active ability. Therefore, it appears especially important to conduct researches on the mechanical properties of injured cartilage. According to the analysis of bone and joint mechanics, a rolling load is the major force among cartilage pressures [1]. In the present study, the noncontact digital image correlation (DIC) technique [24, 25] was used to study changes in the mechanical properties of the articular cartilage related with defect depth under rolling load, in order to summarize the mechanical properties of the injured cartilage. This study may provide a reliable reference for the prevention and the treatment of bone-joint diseases.

## 2. Materials and Methods

### 2.1. Sample Preparation

**Figure 1:**
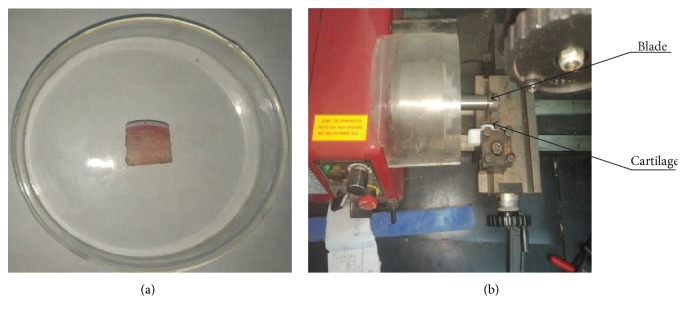
(a) A tested cartilage sample; (b) the picture of making defects.

Fresh knee joint cartilage of the distal femoral end was obtained from a 6-month-old pig. Cartilage slices (length = 8 mm; height = 18 mm; thickness = 3 mm) were cut along the normal direction of the cartilage surface. The defect of cartilage was made of machine tools. The thickness of the circle blade was 0.5 mm, and the circle blade rotated with the main axis. The sample of the cartilage was fixed on the knife rest. The depth of notch was controlled by the feed of knife rest, and the feed precision is 0.1 mm. Notches with a width of 0.5 mm and a varying depth were prepared (Figure 1). Three different notch depths were obtained as follows: 0.2 ± 0.02 mm, 0.5 ± 0.02 mm, and 0.7 ± 0.02 mm which were reached into superficial layer, middle layer, and deep layer, respectively. For each notch depth, 6 samples were prepared and iron oxide nanoparticles, which were treated as pixels, were embedded on the side surface of the slices.

### 2.2. Experimental Device

**Figure 2:**
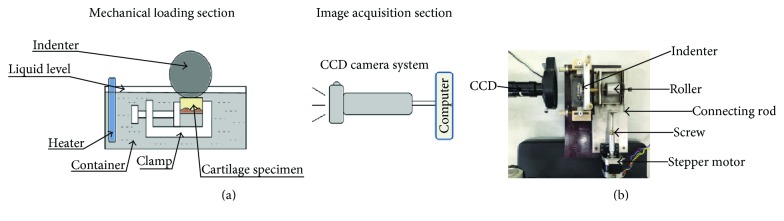
(a) Schematic diagram of the experimental apparatus; (b) the practicality of the experimental apparatus.

Figure 2(a) shows the experimental apparatus which were composed of the mechanical loading system, the image acquisition system, the computer control system, and the image processing software.

Figure 2(b) shows the mechanical loading system including the rolling control device and the compression quantity adjusting device. The rolling control device was driven by a stepping motor, that is, the rotary motion was converted into a linear motion through the screw, then through the connecting rod to drive the cylindrical roller, to thus perform constant reciprocating rolling. The compression quantity adjusting device was regulated by the screw on both sides of the fixture portal frame. This system had a maximum rolling distance of 30 mm and a maximum rolling velocity of 10 mm/s. The diameter of the indenter was 40 mm, and the surface roughness was 0.05. A temperature-controlled liquid tank was implemented on this equipment to simulate in vivo environment.

The image acquisition system mainly consisted of a charge-coupled device (CCD) camera, which helped us obtain images with a 1376 × 1035 resolution. Images were then analyzed and postprocessed by an image processing software, producing data about the displacement and strain of the mark points of cartilage samples.

### 2.3. Experimental Methods

**Figure 3:**
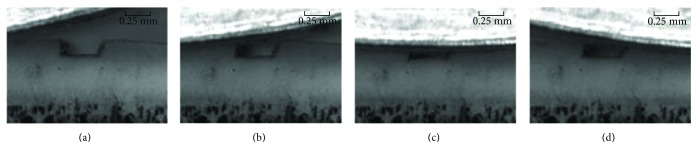
Images acquired by the CDD camera. From (a) to (d) represented image acquisition sequence.

The cartilage samples were fixed on the fixture clamp of the portal frame and then placed in a saline tank. After that, saline was heated to 37°C so as to reduce the experimental errors. The indenter rolled onto the surface of the cartilage sample 50 times back and forth, with a compression quantity of 0.1 mm and a rolling velocity set as 1 mm/s, 2 mm/s, 4 mm/s, and 6 mm/s, respectively. Images were acquired continuously by the CCD camera with 2 frames/s frequency (Figure 3). Then, the displacement and strain fields were obtained after image processing.

## 3. Results

**Figure 4:**
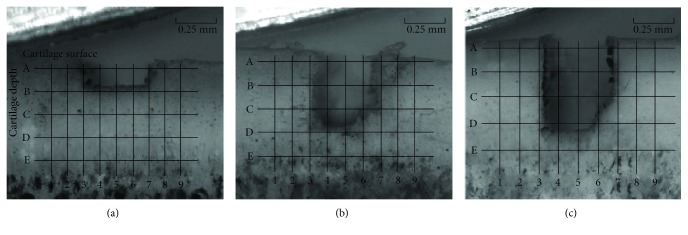
The position of the grid and the grid partition of the different notch depths. (a) Notch depth was 0.2 mm; (b) notch depth was 0.5 mm; and (c) notch depth was 0.7 mm.

In order to facilitate the analysis of stress and strains near the notch, regions near the notch were divided by uniformed grid partition. The interval between two longitudinal lines was set at 0.125 mm. The selected horizontal lines were located 5%, 25%, 45%, 65%, and 85% away from the cartilage surface (Figure 4).

### 3.1. Effect of Defects on the Mechanical Properties of Cartilage

The iron oxide nanoparticles as mark points and pixels were embedded on the side surface of the sample before the experiments. The speckle image of the sample including mark points in its load-free state was first acquired and used as the reference image. The continuous and instantaneous speckle images including the mark points were also obtained at the different stages of loading. The images in random half cycle that the roller rolled above the sample from the left to the right were selected from all the acquired images. Using the computer to identify the mark points and pixels, the displacements of mark points and pixels were calculated by comparing the coordinates of current pixel images with reference image. And then, the strain values were obtained according to the relationship between the displacement and strain.

**Figure 5:**
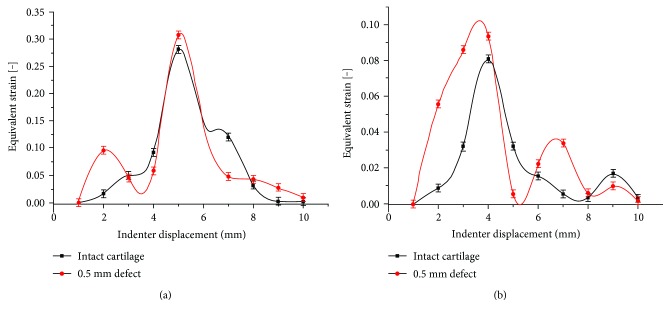
Comparison of the strain curves between intact and defective cartilages. (a) The strain curves at the A3 point; (b) the strain curves at the D3 point.

Figure 5 shows the comparison between intact and injured (notch depth of 0.5 mm) cartilages under a compression quantity of 0.1 mm and a rolling velocity of 4 mm/s. The equivalent strain significantly decreased with the increase of the cartilage notch depth when the roller went through the surface of the cartilage from the left to the right. The cartilage strain values could be changed by defects. Injured cartilage had a larger strain value as well as a higher frequency of strain peak value than the intact cartilage. At the A3 point, which was in the superficial cartilage, the difference of the strain peak value accounted for 6% of the total strain value, while this represented 20% for the D3 point in the deep layer of the cartilage. Results showed that the defect had obvious effects on the mechanical properties of the cartilage.

### 3.2. Effect of Defect Depth on the Mechanical Properties of Cartilage

**Figure 6:**
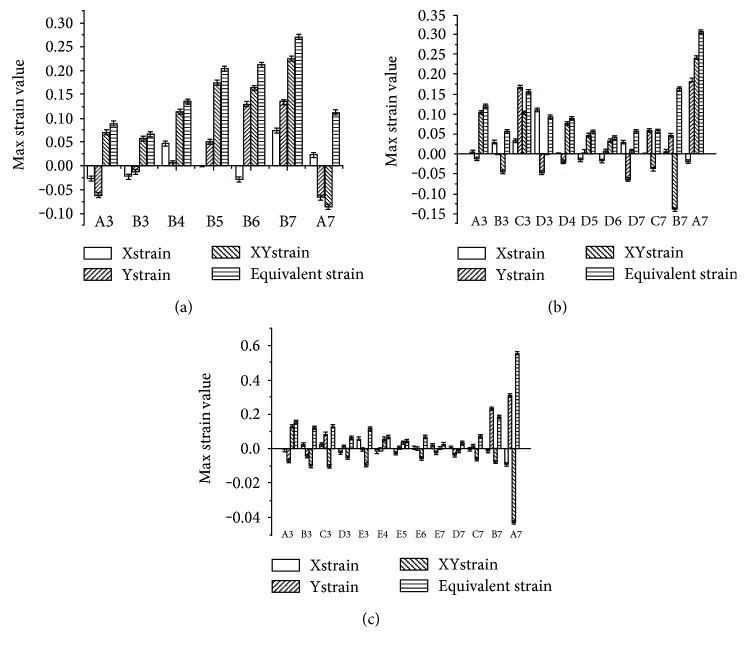
The strain values measured at the observation points around the notches. (a) Notch depth was 0.2 mm; (b) notch depth was 0.5 mm; and (c) notch depth was 0.7 mm.

**Figure 7:**
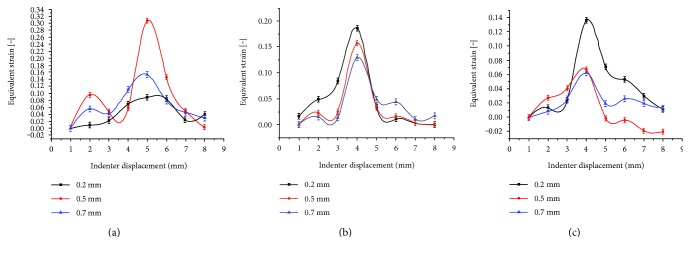
The strain curves measured at different points across the different notch depths. (a) Equivalent strain measured at point A3; (b) equivalent strain measured at point C3; and (c) equivalent strain measured at point D3.

In this part, strain values were measured at each observation point around the notch with different depths under a compression quantity of 0.1 mm and a rolling velocity of 4 mm/s (Figure 6). The cartilage received a periodic load force when the reciprocating roller rolled over the surface of the cartilage, and we selected the maximum strains of the selected points in half cycle for further analysis. It was showed that a shearing strain played a dominant role in the strain value changes. For the cartilage with a notch depth of 0.2 mm (Figure 6(a)), the strain values of the defect's bottom points (B3–B7) gradually increased from the left to the right. Point B7, at the right bottom corner, had the maximum strain value of 0.27, which was 3 times that of the left bottom corner at point B3. For the cartilage with a notch depth of 0.5 mm (Figure 6(b)), the strain values of the bottom points (D3–D7) showed a fall-rise trend from the left to the right. The equivalent strain of the two bottom corners was twice that of the middle region points. For points C7, D7, and C3, the *ε*_y_ strain replaced the shearing strain as the dominate role, and the shearing strain direction changed at points B3 and B7. The strain values at the bottom of the notch (E3–E7) first decreased and then increased from the left to the right when the notch depth was 0.7 mm (Figure 7(c)). For the points at both sides of the notch (B3, C3, D3, A7, B7, and C7), their shearing strain directions changed, and the values gradually decreased with increase of the defect depth.

Figure 7 shows changes in the equivalent strain across the varying defect depth at points A3, C3, and D3 under a compression quantity of 0.1 mm and a rolling velocity of 4 mm/s. For each point, changes in the strain followed a periodic pattern; for the points in the middle and deep layers, the equivalent strain value significantly decreased with the increase in the defect depth; at which the points were located near the superficial layer, the equivalent strain value was very small when damage was relatively small, and the maximum equivalent strain values could be observed with a notch depth of 0.5 mm.

### 3.3. Effect of Rolling Velocity on the Mechanical Properties of Defect Cartilage

**Figure 8:**
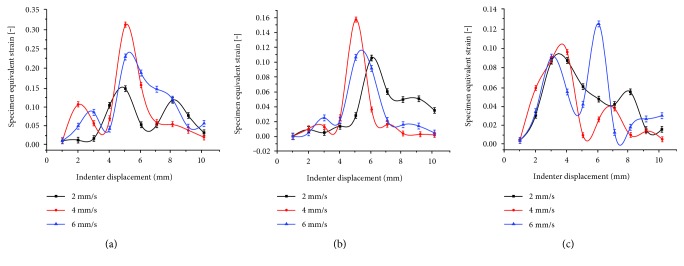
Variation trend curves of strain measured for dangerous points at different rolling velocities. (a) Equivalent strain measured at point A3; (b) equivalent strain measured at point C3; and (c) equivalent strain measured at point D3.

Figure 8 illustrates the fluctuation curves of the equivalent strain at A3, C3, and D3 across different rolling velocities, with a notch depth of 0.5 mm and when the roller moved from the left to the right. For point A3, the minimum peak value was achieved at a rolling velocity of 2 mm/s. At 6 mm/s, the peak value became larger and a maximum of 0.30 was observed at 4 mm/s, which represented 1.5 times of the value measured at the rolling velocity of 2 mm/s.

For point C3, a single distinct peak could be found for the equivalent strain. Peak values presented an increasing trend and then a reduction with the increase in the rolling velocity. A maximum value of 0.16 was observed at a rolling velocity of 4 mm/s, which was 1.5 times of the value recorded at 2 mm/s.

In contrast, the case was rather complicated for point D3. There were two peaks with in half rolling period, and the peak frequency appeared enlarged with the increase in the rolling velocity. The first peak was located in the same position across the different rolling velocities, that is, when the indenter was right above the D3 point, while the second peak was located differently and decreased at velocities of 2 mm/s and 4 mm/s. When rolling at a velocity of 6 mm/s, the second peak increased to 0.12.

## 4. Discussion

In this paper, we used the noncontact digital image correlation (DIC) technique to focus on the mechanical responses of cartilage with different defect depths under rolling load. As the distribution and content of the major components varied with defect depth, fibers inside the cartilage can be divided into three parts: superficial, middle, and deep layers (accounts for 5%, 45%, and 50% of the cartilage thickness, resp.). Collagen fibers in the superficial layer distribute densely and parallel with the articular surface, which has maximum moisture content and for which deformation can easily occur under normal stress. Collagen fibers in the middle layer are irregularly arranged with large interspaces and crisscross at a certain angle with joint surface, which has less moisture content and deforms slowly under normal stress. The deep layer fibers are nearly perpendicular to the articular surface and have the lowest water content and the smallest deformation under normal stress [26]. The method used in our study has the advantages of noncontact, low requirement for the experiment environment and light source, as well as high measurement accuracy, and allows full-field measurement. It has thus been widely used in biomechanical studies [24, 25]. Due to high toughness and different distributions of fibers inside the cartilage, it was very difficult to obtain a smooth border when creating the defect, leading to certain errors in the measurement. In this paper, the left side, which was relatively smooth, was chosen for further analysis in order to reduce errors.

As can be seen from Figure 5, the peak values of the equivalent strain decreased gradually with the increase of the notch depth, which was consistent with the strain law obtained in intact cartilage experiments [22, 27]. When the indenter moved on a cartilage surface from the left to the right, each point was subjected to cyclic loading, leading to cartilage fatigue failure. When a defect existed, the cycle frequency enlarged and the strain value of each point increased around the defect. These results are consistent with those reported by Gratz et al. [14] and are likely due to the stress concentrations produced by the notch. Timely repairing of damaged cartilage can reduce strain value around the notch [28].

When notch is shallow, the maximum equivalent strain appeared at the bottom corner of the notch; with the increase of notch depth, the strain value rose quickly because the supporting structure became loose. Figure 6 showed that the shear strain increased significantly when the notch reached the deep layer, which is in agreement with the results of Dabiri and Li [17] obtained by using a knee joint model. This means that the shear strain of the cartilage increased gradually with cartilage degradation. It can be inferred that the shear strain is the key factor in cartilage destruction, and this destruction began from the bottom corner of the defect.

The positive and negative strain variations at points C3 and D3 existed in our study. These points were located in the vicinity of 50% of the cartilage height, which may be the interface between the middle and the deep layers. The distribution of cartilage fibers may vary in this position, resulting in different strain change rules compared with other points. This result indicated that fiber distribution has an important influence on the mechanical properties of cartilage [29, 30]. The interface between the middle and the deep layers will be easily destroyed when the notch will deepen.

Compared with the other points of the location, the points near the superficial layer showed different responses to the defect depth (Figure 7). Indeed, points located in the superficial layer had greater responses to the defect with a moderate depth, while deep points were sensitive to a small defect. This is considered to be determined by the fiber structure and the viscoelastic properties of the cartilage. Besides, superficial layer has a higher water content, low modulus of elasticity as well as greater deformability. Accardi et al. [31] also confirmed that the fiber orientation of cartilage can resist the shear failure, which indicated that cartilage has a self-protective function against certain damage. This way, the destruction process can be significantly delayed.

The rolling velocity has a certain impact on the cartilage strain. It was reported that the friction coefficient increased first then became lower with the increase in the rolling velocity [32–34], leading to a rising and falling cartilage strain. This may be due to the cartilage viscoelasticity, which ensures that water can be continuously extruded under loading, causing changes in the friction coefficient and the strain values.

The main problem is that the experimental model of defect cartilage was a plane model using digital correlation technology to investigate mechanical properties of defect cartilage. The difference between experimental model and the cartilage model in vivo is that the confining pressure conditions could not be considered. However, the authors think that it is possible to obtain the confining pressure condition of the experimental model by using the method both numerical simulation and experiment. The displacement field and stress strain field of the model of integral cartilage and femur could be obtained by using the numerical simulation method, then the plane model of the experiment was taken into account from the integral numerical model, and the boundary condition was applied to the cutting surface of the plane model base on the results of the integral cartilage and femur. Modified boundary conditions decreased the error between the strains of the experiment and strains of the numerical simulation. The confining pressure conditions could be obtained by the numerical simulation. The confining pressure conditions could be used to conduct the experimental defect cartilage model.

In addition, the analyzed images were from the random half cycle in this paper. Due to the viscoelastic properties of the cartilage, its strain is correlative with rolling number, which could result in experimental errors. As a result of the limitation of cartilage samples taken from the position, the experimental samples from different pig femoral cartilages could also cause error. The physical load of the knee joint involves rolling, sliding, and a combination of rolling and sliding. This paper focused on the defect cartilage subjected to single load such as rolling, and the research work will be carried out in the future to understand the mechanical properties of the defect cartilage under other loads.

## 5. Conclusion

In this paper, we used a noncontact DIC technique to measure the displacement and the strain fields near the notch of a defected cartilage under rolling load. Based on our study, we can conclude that the cartilage damage may increase the strain values and strain peak frequency around the defect. The shear strain, which serves as the main factor causing cartilage destruction, increased with the increase in the defect depth. The cartilage would be destructed firstly at the bottom corner of the defect, and when the defect reached the certain depth, it might be destroyed along the interface between the middle and deep layers. The rolling velocity showed a significant effect on the superficial and middle layers. The equivalent strain increased first and then decreased with the increase in the rolling velocity. Changes were not obvious in the deep layer except for the rising strain peak frequency. The special structure of the cartilage exhibited a self-protective function against destruction, which may slow down this destruction process. Our results can provide a basis for the clinical treatment of osteoarthritis and cartilage repair. It is also of great significance for the mechanical analysis of artificial cartilage.
